# Complex Characterization of Cerebral Vasoreactivity in Internal Carotid Artery Stenotic Patients with Transcranial Doppler Sonography

**DOI:** 10.3390/life15111692

**Published:** 2025-10-30

**Authors:** Hanga Pál, Rita Magyar-Stang, Borbála Csányi, Anna Gaál, Zsuzsanna Mihály, Zsófia Czinege, Péter Sótonyi, Tamás Horváth, Balázs Dobi, Dániel Bereczki, Akos Koller, Róbert Debreczeni

**Affiliations:** 1 Department of Neurology, Semmelweis University, Balassa Str. 6, H-1083 Budapest, Hungary; pal.hanga@semmelweis.hu (H.P.); stang.rita@semmelweis.hu (R.M.-S.); gaal.anna@semmelweis.hu (A.G.); bereczki.daniel@med.semmelweis-univ.hu (D.B.); 2 János Szentágothai Neurosciences School of PhD Studies, Semmelweis University, H-1085 Budapest, Hungary; csanyiborbala10@gmail.com; 3 Department of Vascular and Endovascular Surgery, Semmelweis University, H-1122 Budapest, Hungary; zsuzsanna.mihaly@semmelweis.hu (Z.M.); czinege.zsofia@gmail.com (Z.C.); sotonyi@hotmail.com (P.S.); 4 Research Center for Sport Physiology, Hungarian University of Sports Science, H-1123 Budapest, Hungary; horvath2.tamas@tf.hu (T.H.); akos.koller@gmail.com (A.K.); 5 HUN-REN Neuroepidemiological Research Group, H-1117 Budapest, Hungary; balazs.dobi@gmail.com; 6 Cerebrovascular and Neurocognitive Disorders Research Group, Translational Medicine Institute, Faculty of Medicine, and HUN-REN-SE, Semmelweis University, H-1094 Budapest, Hungary; 7 Department of Physiology, New York Medical College, Valhalla, NY 10595, USA

**Keywords:** transcranial Doppler sonography, carotid artery diseases, cerebral vasoreactivity, atherosclerosis

## Abstract

Background and Aims: Decreased cerebrovascular reactivity (CVR) in patients with significant internal carotid artery stenosis (ICAS ≥ 70%) is an independent risk factor for cerebral infarction. To evaluate CVR, changes in cerebral perfusion pressure and blood flow velocity (BFV) of the middle cerebral artery (MCA) can be estimated by CO_2_- (hyperventilation—HV and breath-holding—BH) and pressure–flow-based (Common Carotid Artery Compression—CCC and Valsalva Maneuver—VM) stimuli. We used a multimodal approach to characterize CVR in patients before carotid endarterectomy (CEA). Methods: HV, BH, CCC, and VM tests were performed on 31, 26, and 34 patients. BFV of MCAs was monitored by transcranial Doppler, and continuous arterial blood pressure was registered non-invasively. CVR was compared between the operated significantly stenotic and the contralateral sides. Results: The extent of HV- and BH-induced CVR was similar, but the time to the lowest HV-induced BFV was shorter on the side with significant ICAS. The response to CCC was sensitive to hemodynamic asymmetry in the transient hyperemic response ratio and in the cumulative change in the (mean arterial blood pressure)/(mean BFV) ratio. In VM, the slope of BFV increased in the ascending (2b) phase, and the time to overshoot correlated with the side of the stenosis. Conclusions: These results suggest that in patients with significant ICAS, in addition to CO_2_ reactivity measurements, a more complex estimation of CVR, by using hemodynamic tests (CCC and VM), should also be used to better quantify cerebral ischemic risk.

## 1. Introduction

To maintain a proper and adequate cerebral blood supply, cerebral vasoreactivity (CVR) is essential for the brain. This means rapid and significant reactions of the cerebral arterial vessels, also known as resistance vessels, which protect the brain tissue from hyper- and hypoperfusion by altering their diameter [1,2]. CVR is controlled by several different mechanisms, often acting simultaneously [3,4]. These vascular responses are elicited by changes in perfusion pressure and flow, blood carbon dioxide partial pressure (pCO_2_), and increases in local neural activity by neurovascular coupling [1]. Changes in the diameter of the resistance vessels results in changes in blood flow to the brain tissue, which is associated with a proportional change in the blood flow velocity in the large cerebral arteries. Transcranial Doppler ultrasonography (TCD) is an easy-to-use, inexpensive, and non-invasive technique, which is an ideal tool for estimating cerebrovascular reactivity as a function of time, by measuring changes in blood flow velocity in large cerebral arteries to different stimuli [5,6,7,8,9,10,11,12].

Atherosclerosis frequently develops in elderly population, affecting the blood circulation of nearly all organs, and thus the blood supply of the brain as well. Indeed, significant atherosclerotic stenosis in the internal carotid artery (ICAS, ≥70%) is responsible for approximately 10–20% of cerebral ischemia/infarctions [13]. It is known that severe ICAS can lead to impaired CVR [14], which is an independent risk factor for cerebral infarction as described in the 2023 European Society for Vascular Surgery (ESVS) Guidelines [15]. In patients with advanced carotid artery atherosclerosis, reduced cerebral perfusion (pressure and flow) can develop due to exhaustion of the capacity of cerebrovascular reactivity [16,17,18,19].

Thus, the aim of the present study was to characterize cerebrovascular reactivity in a complex manner as a function of the severity of ICAS in patients before undergoing carotid reconstruction surgery, which helps to a better and more comprehensive preoperative estimation of cerebral ischemic risk [20].

The impairment of CVR because of severe ICAS has already been examined in several previous studies using different approaches [14,21,22,23,24,25,26,27]; therefore, we decided to examine a patient population with a complex TCD protocol.

### 1.1. Reactivity of Cerebrovascular System to Changes in Blood CO_2_ Concentration

Reactivity of cerebral vessels to CO_2_ is the phenomenon when arterioles respond to changes in arterial partial pressure of carbon dioxide (pCO_2_) between 20 and 60 mmHg [28]. An increase results in the dilation of cerebral resistance vessels with consequent increase in cerebral blood flow, whereas a decrease in pCO_2_ induces vasoconstriction and thus decrease in cerebral perfusion [28,29,30,31,32,33]. In humans, changes in arterial pCO_2_ can be elicited by moderate hyperventilation (HV) and breath-holding tests (BH), which are self-controlled and therefore safe [7,34]. During hyperventilation, the pCO_2_ concentration of arterial blood decreases, which causes vasoconstriction of cerebral arterioles [35]. Vice versa, during breath-holding, the arterial pCO_2_ increases, which leads to the dilation of the cerebral resistance vessels. The subsequent changes in the mean blood flow velocity (MBFV) in the middle cerebral artery (MCA) can be measured by using TCD [7,8,34,36,37,38].

### 1.2. Pressure–Flow Reactivity

The pressure–flow reactivity of cerebral resistance vessels, which also characterizes cerebral vasoreactivity, can be measured in several ways.

#### 1.2.1. Common Carotid Artery Compression (CCC) Test

One of them is the common carotid artery compression (CCC) test, a vasoactive stimulus that reflects the autoregulatory integrity of cerebral vessels [9,10,11,39,40]. During the CCC test, a manual compression on the common carotid artery is performed for several seconds, which results in a temporary decrease in perfusion in the ipsilateral MCA. Because of the compression, the decreased MCA perfusion results in the dilation of small cerebral vessels of that MCA territory to maintain an adequate blood supply to the brain tissue. After carotid compression, BFV transiently increases, indicating reactive hyperemia (THR) and is likely the result of a decrease in blood flow resistance induced by cerebral hypoperfusion during carotid compression. The CCC test allows for calculating the transient hyperemic response ratio (THRR), which indicates the “normalized” changes in blood flow velocity to changes in the cerebral perfusion pressure [11]. The CCC test is also an accepted test for modeling the effect of carotid clamping during surgical interventions; thus, this test is relevant to quantify CVR in these patient populations [24,41].

#### 1.2.2. Valsalva Maneuver (VM)

Valsalva Maneuver (VM) is another standardized test providing physiological stimuli evoking considerable changes in the central arterial blood pressure during forced exhalation against a resistance (due to closed glottis or a valve). The analysis of cerebral blood flow velocity response to mean arterial blood pressure (MABP) changes is suitable for estimating cerebrovascular reactivity, whereas changes in blood pressure and heart rate indicate the regulation of cardiovascular functions by the autonomic nervous system [42,43,44,45,46,47,48,49]. During VM, characteristic changes occur in cerebral MBFV, MABP, and HR, which can be divided into four phases as a function of time [42].

Phase I: At the onset of the VM, the increase in intrathoracic pressure is transmitted to the vascular system, thereby increasing central venous pressure and MABP.

Phase IIa (early): The maintained increased intrathoracic pressure reduces venous return to the heart, resulting in a decrease in the right and then in the left ventricular stroke volume, and thus MABP progressively decreases.

Phase IIb (late): A decrease in MABP activates the sympathetic nervous system, which regulates the cardiovascular system, which causes an increase in peripheral vascular resistance and heart rate, leading to an increase in MABP.

Phase III: When forced exhalation against resistance is stopped, there is a sudden drop in the intrathoracic pressure, resulting in a rapid and temporary decrease in MABP.

Phase IV: A fast and significant rise in the MABP and HR in several seconds is observed due to the previously elevated peripheral vascular resistance and the increased venous return (overshoot; OS). Following the OS, reflex bradycardia occurs due to the activation of the baroreflex, and finally, MABP and HR return to baseline.

## 2. Materials and Methods

This cross-sectional, single-center, exploratory study was approved by the Semmelweis University Regional and Institutional Committee of Science and Research Ethics (SE RKEB, Number: 256/2018) and registered at ClinicalTrials.gov (Identifier: NCT03840265). The study was conducted according to the guidelines of the Declaration of Helsinki. Patients admitted to the Department of Vascular Surgery of Semmelweis University for reconstruction of ICAS were enrolled consecutively between 15 January 2019 and 30 September 2021 after providing written consent. The severity of ICAS was determined by Computer Tomography Angiography according to the North American Symptomatic Carotid Endarterectomy Trial (NASCET) criteria, with a stenosis of 70% or greater considered hemodynamically significant (i.e., flow-limiting stenosis) [50,51].

In the present study, patients were enrolled with significant (≥70%) and tend-to-be-operated ICAS—ICA_op_, regardless of the condition of the contralateral side—not-to-be-operated—ICA_nonop_, which was <70% or ≥70%. Exclusion criteria were suboptimal transtemporal insonation window, atrial fibrillation, pacemaker therapy, and extensive atherosclerotic plaques in the CCA. The examinations were performed at the Doppler Laboratory of the Department of Neurology at Semmelweis University. Patients were continuously monitored clinically during the investigations. Demographic and medical history data were collected. To achieve reproducibility and best signal-to-noise ratio, the CCC and VM were performed three times, and the largest response was evaluated for each patient. The study group consisted overall of 48 eligible patients (male: 36 mean age ± SD: 68.08 ± 7.23 years; female: 12 mean age ± SD: 70.16 ± 7.35 years). The risk comorbidities of the patients were as follows: 44 patients had hypertension (91.7%), 21 of them had diabetes mellitus (43.8%), 16 were smokers (33.3%), and 14 patients had ischemic heart disease (29.2%). The patients were asymptomatic regarding the ICAS and received antiplatelet therapy in addition to antihypertensives and oral statin medication. The series of vasoactive tests used took an average of one hour. All stimuli were safe, none of the patients tested reported any discomfort, and no neurological complications were experienced. The patients’ cooperation was excellent in all tests, and the VM was performed well after 1–2 attempts.

### 2.1. Transcranial Doppler (TCD) Study Protocol

As previously described [25,26], the blood flow velocity (BFV, cm/s) in both MCAs was recorded by TCD probes (2 MHz, DWL Multi-Dop T2, Sipplingen, Germany) in a semi-sitting position through the transtemporal window at a depth of 45–55 mm. During TCD measurements, continuous, non-invasive, beat-to-beat arterial blood pressure (ABP) monitoring was performed by radial artery applanation tonometry (Colin-BP508, Hayashi Komaki Aichi, Japan). Heart rate was continuously monitored with electrocardiogram (ECG).

During the examination of patients, 3 vasoactive stimuli were applied in the following order:

1. HV/BH test
2. CCC test
3. VM

#### 2.1.1. HV/BH Test

We used a combined stimulus to assess the CO_2_ reactivity of cerebral vessels, with the aim of evaluating the maximal CO_2_-dependent cerebral vascular response; therefore, we chose voluntary hyperventilation and breath-holding as a simple, safe, and well-tolerated maneuver [7,34,37]. In basal conditions (baseline, BL), parameters measured were recorded after 15 min adaptation period. After BL, patients performed the HV test for 30 s in a semi-sitting position and were asked to inhale deeply and exhale dynamically. At the beginning of the HV test, MCA MBFV values started to decrease until they reached their minimum value. Then, 30 s later, patients were asked to inhale and hold their breath as long as they could. During the BH test, MBFV values started to increase and reached their maximum. Patients raised their hands when they finished the BH test. Each patient had a different BH test time (average 42.3 s, SD: ± 21.67). A total of 31 patients met the best signal-to-noise ratio requirements (74.2% male, age: 68 ± 5.11 years, and 25.8% female, age: 71.5 ± 4.89 years).

#### 2.1.2. CCC Test

Before defining the study protocol, we carefully reviewed previous publications regarding CCC tests and noticed that no clinical complications have been reported [10,11,52,53]. Since previous publications confirmed that 10 s were required to achieve the maximal CCC BFV response, we used 10 s of manual compression of the CCA [10]. During the maneuver, bilateral MCA blood flow velocity values were measured with TCD. The CCC test was performed on both sides, one by one, after the CCA status was previously checked with duplex Doppler ultrasound. In case of extensive CCA atherosclerosis, the carotid compression test was omitted due to the risk of embolism. Compression was performed 3 times on both CCAs, with a 2 min interval between each maneuver. For the statistical analysis, the technically best and largest amplitude reactions were selected. A total of 26 patients met the best signal-to-noise ratio criteria (76.9% male, age: 68.67 ± 8,6 years, and 23.07% female, age: 71.17 ± 0.22 years).

#### 2.1.3. VM

The duration of the VM was standardized to 15 s. VM was performed with forced exhalation against a sphygmomanometer by maintaining their expiratory pressure at 40 mmHg, which was controlled by the patient and the assistant as well. Recordings of those patients were used, which had an adequate signal-to-noise ratio and were thus suitable for complex signal analysis. The 34 patients’ VM was evaluated (76.5% male, age: 66.96 ± 7.34 years and 23.5% female, age: 70.62 ± 5.59 years).

### 2.2. Data Processing

Digitization of analog signals was performed in parallel on four channels (TCD1, TCD2, ABP tonometry, ECG), with a sampling rate frequency of 500 Hz. Raw data were stored in the European Data Format files. The files were imported, digitally filtered, and segmented using the LabChart software (ADInstruments, LabChart ver. 8, Colorado Springs, CO, USA). The pre-processed data segments describing each cardiac cycle were stored in separate text files. These files were further processed by custom-written Python code (Python version 3.12.2). The program script interpolated cardiac cycle data linearly into 0.5 s equidistant intervals. The interpolated data were exported to Microsoft Excel, and the changes in systolic, mean, and diastolic BFV, ABP, HR, and various vascular reactivity parameters were calculated.

### 2.3. Variables

#### 2.3.1. HV and BH Tests 

**Figure 1:**
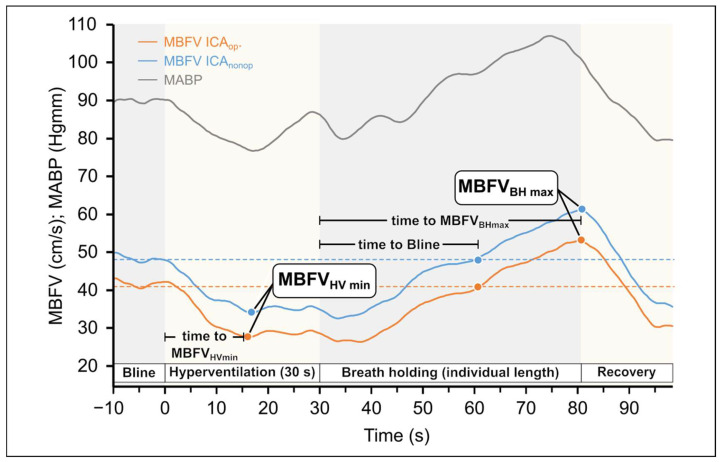
Original records of changes in the mean blood flow velocities (MBFV) measured in the middle cerebral artery (MCA) in patients with unilateral carotid stenosis during hyperventilation and breath-holding tests. The MBFV decreased during hyperventilation, whereas it increased during breath holding test, indicating that changes in blood CO_2_ level elicited the expected changes in cerebrovascular resistance. The blue line indicates the side of ICA_nonop_, whereas the yellow line indicates the side ICA_op_. The minimum and maximum values of MBFV are indicated together with time characteristics. It can be seen that during the whole response, the MBFV was substantially lower (with about 10 mm/s) in the severe stenotic side compared to the other side and followed the changes in blood pressure. The changes in mean blood pressure are indicated with a gray line.

Variables of the two stimuli are summarized in Table 1. Figure 1. shows the flow chart of the HV and BH tests.

#### 2.3.2. Cerebral Arterial Resistance (CAR) Values During HV/BH Test

**Figure 2:**
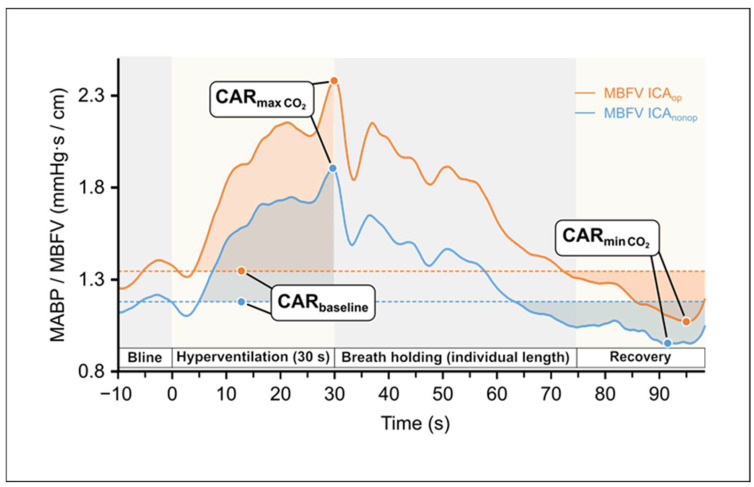
Changes in the calculated cerebral arterial resistance (CAR: MAPB/MBFV) of both hemispheres during hyperventilation test as a function of time indicate cerebral vasoconstriction and during breath-holding test indicate cerebral vasodilation, respectively. The areas with darker colors represent the area under the curve (Data used from Figure 1).

Cerebral arterial resistance was defined by the ratio of systemic mean arterial blood pressure (MABP) and mean cerebral blood flow velocity in the middle cerebral artery (MBFV) according to the formula [25,54,55] (see Figure 2):

CAR = MABP/MBFV.

The variables regarding CAR in HV/BH tests are summarized in Table 2.

#### 2.3.3. Cerebral Arterial Resistance Area Under the Curve (CAR-AUC) Calculations During HV/BH

The baseline CAR for HV/BH tests was determined by averaging the CAR values over the 10 s preceding the stimulus. Actual CAR values were subtracted from baseline (CAR_bl_—CAR_actual_), and the cumulative change in CAR until return to baseline CAR was determined by calculating the area under the curve (AUC) using the trapezoidal method (see Figure 2).

$$\mathrm{CAR}-\mathrm{AUC}=\sum_{i=1}^{n}\frac{{CAR}_{i-1}+{CAR}_{i}}{2}\times 0.5$$

#### 2.3.4. CCC Test

The defined variables of the CCC test are summarized in Table 3.

**Figure 3:**
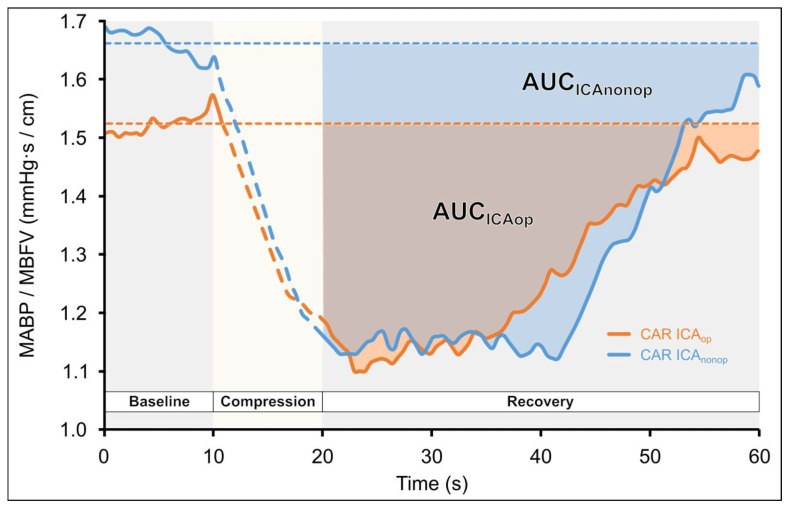
Changes in the calculated cerebral arterial resistance (CAR: MAPB/MBFV) during common carotid artery compression (CCC) test in a patient with unilateral carotid stenosis as a function of time. Carotid compression resulted in cerebral hypoperfusion, which elicited dilation in the arterial vessels supplied by the middle cerebral artery (MCA); thus, CAR decreases. After the compression is released, the CAR gradually returns to baseline. Area under the curve (AUC) was calculated by the trapezoid method until the baseline-subtracted CAR values returned to zero. The areas with darker colors represent the area under the curve. It can be seen that AUC of ICA_op_ is smaller than the contralateral one.

CAR_CCCAUC_: The baseline CAR for CCC tests was determined by averaging the CAR values over the 10 s preceding the stimulus. CAR could not be calculated during carotid compression because the perfusion pressure required to maintain MCA MBFV could not be measured. After carotid compression was released, actual CAR values were subtracted from the baseline (CAR_BL_—CAR_actual_), and the cumulative change in CAR until return to baseline CAR was determined by calculating the area under the curve (AUC) using the so-called trapezoidal method mentioned before. The CAR AUC of the ICA_op_ and ICA_nonop_ sides was compared statistically (see Figure 3).

$$\mathrm{CAR}-\mathrm{AUC}=\sum_{i=1}^{n}\frac{{CAR}_{i-1}+{CAR}_{i}}{2}\times 0.5$$

#### 2.3.5. VM

**Figure 4:**
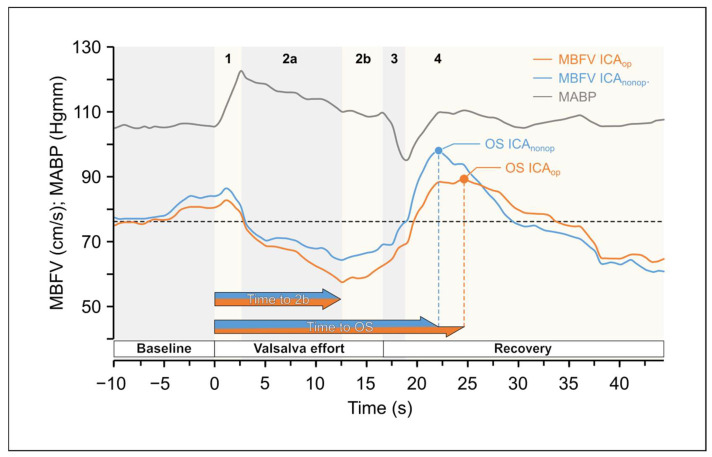
Original records of changes in the mean blood flow velocities (MBFV) measured in the middle cerebral artery (MCA) in patients with unilateral carotid stenosis during Valsalva Maneuver (VM) as a function of time. There were considerable changes in the mean arte-rial blood pressure (MABP). yet Yet changes in MBFV in the middle cerebral arteries (MCAs) were different in the two sides. Changes were divided into four phases: there was side difference in MBFVdifference of MBFV values during phase 2a and 2b and a time shift in maximalshift of maximal values between MBFV overshoot (OS) in phase 4. The changes in mean blood pressure are indicated with graygrey line.

The variables of the VM are summarized in Table 4. Figure 4. shows the changes in the MBFV and MABP elicited by VM (see the detailed description in Figure 4. legend).

### 2.4. Statistical Analysis

Statistical analysis was performed with TIBCO Statistica^®^ 13.5.0 program and the R programming language version 4.4.1. Data were visualized using Inkscape 1.4. Software and the R programming language version 4.4.1. The significance level was defined as *p* < 0.05 for all tests.

To examine the impact of ICAS on CVR, the variables of the four stimuli of the side to be operated on (ICA_op_) and not to be operated on (ICA_nonop_) were compared. Additionally, grouped comparisons were also performed by dividing the side not to be operated into two groups:

Subgroup 1: Where the stenosis of the ICA was estimated to be equal to or above 70%.

Subgroup 2: A group where the stenosis was less than 70%.

The Wilcoxon rank test was used as the main tool due to the low sample size and non-normal distribution of variables. Since almost all time-to-event variables were free from censoring, they were also compared using the above tests; however, they were evaluated using clustered Cox proportional hazards models, too.

## 3. Results

### 3.1. HV and BH Tests

Original records of variables and the hyperventilation protocol are shown in Figure 1. Figure 1. illustrates the changes in the MBVF and the MABP during the HV and BH tests. During HV in both MCA, a decrease in MBFV can be observed due to the vasoconstriction of the cerebral vessels triggered by the consequently reduced arterial CO_2_ levels. In contrast, the MCA MBFV values increase during the breath holding because of vasodilation. 

Figure 2 presents the CAR changes elicited by the mentioned vasoconstriction and vasodilation; CAR values increase during HV and decrease during BH. Summary data of this test are shown in Table 5.

The data show that the time to MBFV_HVmin_ was significantly shorter on the ICA_op_ side (Wilcoxon ranked test *p* = 0.012) compared to the contralateral side. According to subgroup analysis, the difference was only justified if the stenosis on the ICA_nonop_ side did not reach 70% (Wilcoxon ranked test *p* = 0.0088).

In response to the BH test, original record shows no differences in the response of the two MCA sides. Data in Table 5 indicate that no significant differences were found between the defined indices, except in the subgroup 2 analysis regarding ΔMBFV_BH_ (*p* = 0.042).

To assess the changes in the resistance of cerebral arterial vessels during these tests, CAR values were calculated as well. Data included in Table 1 show no significant differences regarding the CAR values in the two MCA sides (Table 1).

### 3.2. CCC Test

During CCC, a reduction in MBFV is observed in the ipsilateral MCA due to the compression, which is followed by a consequent transient hyperemic response after the release of the compression. Figure 3. presents the CAR-AUC calculations during the CCC test.

The statistical data obtained in this test are included in Table 6. The BL values of the PSV were significantly different between the two sides; thus, relative values were quantified. The value of THRR_PSV_ was significantly higher on the ICA_nonop_ side (Wilcoxon ranked test *p* = 0.0046 and *p* = 0.0033 according to the subgroup 2 analysis). It has also been verified previously that the PSV values might be more sensitive when quantifying the CCC test [52].

The calculated arterial resistance, CAR_CCCAUC_, was significantly lower on the ICA_op_ side (Wilcoxon ranked test *p* = 0.021 and *p* = 0.039 according to the subgroup 2 analysis) compared to the ICA_nonop_ side.

Values are mean ± SD using Wilcoxon ranked test (*p* < 0.05). Significant differences are indicated in bold letter.

### 3.3. VM Test

The statistical essences of the VM are the following:

(1) CVAR had a significantly lower value on the ICA_op_ side. (Wilcoxon ranked test *p* = 0.002 and *p* = 0.014 according to the subgroup 2 analysis).
(2) OS time was significantly longer on the ICA_op_ side (both according to Wilcoxon ranked test with *p* = 0.015; *p* = 0.038 according to the subgroup-2 analysis) and the Wald test of the Cox proportional hazards model with *p* < 0.0001.
(3) Evaluating the autonomic values, we found the following:
  - 15 patients had a VHRR value less than 1.35;
  - 8 patients had a longer PRT than 4 sec;
  - 23 patients had an SI underneath 0.

The statistical data obtained in this test are included in Table 7.

## 4. Discussion

The novel aspect of our study was the use of a comprehensive protocol to assess cerebrovascular regulatory impairments in patients with significant internal carotid artery stenosis. This protocol combined evaluations of changes in blood pCO_2_, hemodynamic forces, and autonomic nervous system, using transcranial Doppler method to measure middle cerebral artery blood flow velocity responses to several physiological tests.

The present study provided several important findings in ICA stenotic patients:

1. CO_2_ changes induced by hyperventilation and breath-holding elicited similar responses in MBFV on both carotid/MCA sides (i.e., ICA_op_ and ICA_nonop_). The only significant differences were found between the two sides in the time to reach the minimum MBFV of the ipsilateral MCA induced by hyperventilation and ΔMBFV_BH_ in subgroup 2.
2. CCC elicited different reactive hyperemic blood flow velocity responses in the two carotid/MCA vascular regions. Two indices of response to CCC were different: a) the THRR index calculated from MCA peak systolic blood flow velocity values and b) the cumulative change in cerebral arterial resistance (CAR-AUC) after cessation of common carotid artery compression. The two mentioned variables were significantly lower on the planned operated side (ICA_op_) compared to the other side (ICA_nonop_).
3. Valsalva Maneuver induced different flow changes in the two carotid/MCA sides. Calculated resistance index, CVAR, and the time to OS were significantly reduced on the side to be operated on (ICA_op_) compared to the other side (ICA_nonop_).
4. Changes in systemic arterial blood pressure and heart rate in response to VM indicated that most patients also had severe cardiovascular autonomic dysfunction.

### 4.1. Clinical Importance of Our Findings

#### 4.1.1. CO_2_ Reactivity (Hyperventilation/Breath-Holding)

In the cerebral vasoreactivity studies included in a meta-analysis, CVR was determined based on changes in cerebral blood flow velocity induced by increasing blood CO_2_ concentration or intravenous administration of acetazolamide, evaluating the maximal cerebral vasodilation capacity [27].

In the protocol of the present study, using the hyperventilation (HV) and breath-holding (BH) tests, the total CO_2_ reactivity was calculated in addition to the previously recommended indices. This represents our novel approach for evaluating the overall CO_2_ reactivity of cerebral resistance vessels.

In the patient population studied, no difference was found in the magnitude of CO_2_-decrease–induced reactivity between the ICA_op_ and ICA_nonop_ sides, but the minimum of the MBFV occurred earlier on the ICA_op_ side. This difference can indicate reduced cerebral vasoconstriction capacity on the ICA_op_ side.

It is of note that the BH index calculated from the MBFV data of patients showed a higher value than that of healthy individuals of the same age reported in a previous publication [8]. This is probably due to the fact that during the BH test, the arterial blood pressure of patients involved in our study significantly increased, and in parallel, their cerebral blood flow velocity increased partially, as a function of systemic blood pressure, indicating an impairment of the CVR. Thus, we recommend that the simultaneous change in arterial blood pressure should be considered when establishing the BH index and that the CO_2_ vasodilation reactivity be characterized by the resistance index calculated in this way (Figure 2).

#### 4.1.2. Pressure–Flow Cerebral Vasoreactivity

The response of cerebral arterial vessels to changes in cerebral perfusion pressure/flow was assessed by the CCC test and the Valsalva Maneuver.

##### CCC Test

The CCC test estimates the effectiveness of cerebral pressure–flow regulation by temporarily reducing the ipsilateral MCA perfusion [9]. If, during CCC, the 10 s hemispheric cerebral low perfusion reaches a critical level, focal deficit symptoms may appear [52], but we did not observe this in the present study, confirming the safety of the CCC test.

In response to CCC, the calculated THRR_PSV_ index was significantly lower on the ICA_op_ side. During compression, ipsilateral MCA perfusion was reduced which was indicated by the decreased MBFV. This elicited (passive and active) vasomotor response of cerebral resistance vessels, indicated by the cerebral arterial resistance change. After the compression was released, a transient increase in MCA MBFV was observed, and then CAR was lower than the baseline value preceding CCC, which may be due to the dilation of cerebral resistance vessels of MCA territory. The cumulative change in the cerebral arterial resistance (CAR_CCCAUC_) was significantly smaller on the side of the ICA_op_ side, suggesting that the dilation capacity of resistance vessels was reduced. Thus, the index which was introduced in the present study seems to be sensitive to hemodynamic disturbances resulting from severe carotid stenosis (Figure 3).

##### VM Test

To assess the VM-test–induced cerebrovascular reactivity, several indices have been published previously [17,46,49,56]. In the present study, CVAR (rate of increase in MCA MBFV in phase 2b) was significantly lower on the ICA_op_ side. In addition, there was a difference in the OS time, because OS occurred later the ICA_op_ than on the contralateral side; thus, this new temporal variable may also be a suitable index for characterizing the dynamics of cerebrovascular reactivity (Figure 4).

The Valsalva Maneuver allows to assess not only cerebral pressure–dependent vasoreactivity but also the regulation of arterial blood pressure and heart rate by the autonomic nervous system. The values of specific cardiovascular autonomic variables of the Valsalva Maneuver suggest that the majority of patients studied also had cardiovascular autonomic dysfunction, which, in addition to impaired cerebral vasomotor regulation, may further increase the risk of cerebral ischemia, especially during potential hypotension during carotid reconstruction surgery/intervention [58]. In these cases, we thus consider VM as an important, overall comprehensive hemodynamic and cardiovascular autonomic test. In these cases, we recommend expanding intraoperative arterial blood pressure and heart rate monitoring with simultaneous and continuous middle cerebral artery TCD recording.

### 4.2. Why Is It Necessary to Use Several Tests and a More Complex TCD Protocol?

In clinical practice, the most widely used and proven methods for determining cerebrovascular reactivity are the CO_2_ reactivity tests and the breath-holding index. However, when this index is calculated, changes in arterial blood pressure are not taken into consideration, since, based on the prevailing assumption, no significant hemodynamic changes occur during the test. However, the data of the present study showed that changes in arterial blood pressure are not negligible during these tests. Thus, changes in cerebral blood flow velocity must be “normalized” to changes in arterial blood pressure to obtain a correct evaluation of changes in CVR, which would be very important to develop a more proper preoperative ischemic risk assessment.

The patients involved in the present study had severe carotid artery stenosis, which can cause reduced cerebral vasoreactivity, and were awaiting reconstructive carotid vascular surgery. During the operation, the carotid arteries are clamped, eliciting reduction in brain perfusion (intraoperative cerebral hypoperfusion). That is why we added hemodynamic stimuli to the preoperative assessment of cerebrovascular reactivity tests.

The results of the measurements and detailed analysis of data confirmed that the CCC and VM tests, which induce significant changes in pressure/flow in the cerebral arteries, were different between the two sides, providing important information regarding the preoperative cerebral ischemic risk.

The VM and CCC tests are eliciting fast and considerable hemodynamic changes, i.e., the levels of blood pressure and flow to the brain. In contrast, during the HV/BH test, cerebral hemodynamic conditions remain relatively stable; thus, changes in cerebrovascular reactivity are predominantly due to changes in arterial CO_2_ concentration. As we have shown, almost all members of our patient population were hypertensive; in addition, they had other diseases, such as diabetes, and harmful habits like smoking, which may have also impaired cerebrovascular function. In the present study, according to the data of comparative statistical analysis, we conclude that CO_2_ sensitivity of cerebral vessels is maintained even in severe ICA stenosis; thus, an assessment based only on CO_2_ tests does not provide sufficient information about the hemodynamic effects of severe carotid stenosis.

In other words, the HV and BH tests do not provide sufficient information regarding the effects of carotid stenosis on the vasomotor capacity of cerebral circulation regulation in a significant proportion of patients. In the present study, however, the responses to hemodynamic changes elicited by CCC and VM tests were prolonged on the ICA_op_ side compared to the ICA_nonop_ side. Thus, we propose that inclusion of CCC and VM tests in the evaluation of patients is important to provide a more complex assessment of cerebrovascular reactivity in patients with ICA stenosis. Overall, a complex testing protocol that includes tests based on pressure–flow changes in addition to CO_2_ reactivity tests might be beneficial.

### 4.3. Study Limitations

One of the limitations of our study was the relatively low number of patients mainly due to the lack of adequate insonation window. In addition to proper insonation window requirements, we only used the bilateral TCD measurements for the statistical analysis, which also limited the number of patients examined. Moreover, due to our strict set of criteria, we found the quality of many measurements to be inappropriate, and despite the initial relatively high numbers of patients examined, the statistical measurements were performed on a relatively smaller number of measurements. Another disadvantage is that the duration of the BH test cannot be fully standardized, because not all patients were able to complete the task in accordance with the instructions. In the future, we will consider standardizing the duration of the BH test. We were unable to calculate the CAR AUC value of the HV test in one patient due to movement artifact, and similarly, in 11 cases, the CAR AUC value of the BH test could not be calculated because the CAR value did not exceed the BL level. Our method for assessing cerebral vascular CO_2_ reactivity lacked end-tidal CO_2_ measurement, which would have facilitated comparative analysis of the data despite different breath-holding times. This may explain why, in contrast to previous publications, we did not observe a significant decrease in CO_2_ reactivity on the side of carotid stenosis.

Furthermore, in an ideal situation, one could have checked the hemodynamic and cerebral vascular reactivity consequences of the ICAS by Single Photon Emission Computer Tomography; however, at the time of the present study, it was not available to us due to the restrictions during the COVID-19 pandemic.

### 4.4. Strength of the Study

Our study summarizes the tests previously considered suitable for assessing CVR and presents it on a relevant patient population. The combination of the vasoreactivity tests utilized allows complex hemodynamic characterization and even more personalized decision-making. This aspect could have implications for ICA-stenotic patients’ preoperative risk assessment in future studies.

### 4.5. Translational Aspects

The findings of the present study have high translational values as they were obtained in humans; thus, in clinical settings measuring, these parameters would allow personalized diagnosis regarding cerebrovascular status and systemic hemodynamics and provide evidence to further develop the guidelines.

## 5. Conclusions

A significant finding of the present study is that in patients with significant carotid artery stenosis the CO2 reactivity tests were not sufficiently sensitive to hemodynamic disturbances caused by significant carotid artery stenosis. Thus, other tests, such as hemodynamic tests (CCC and VM), which elicit changes in cerebral perfusion pressure and flow, should be used to assess cerebrovascular reactivity and quantify cerebral ischemic risk. Thus, it is important to assess cerebrovascular reactivity in a complex manner. However, we recommend further prospective studies to assess whether this complex preoperative risk assessment method can be recommended in clinical practice.

## Figures and Tables

**Table 1:** Summary of the variables of the hyperventilation and breath-holding tests.

Definition of Indicator	Abbreviation	Description
Baseline Mean Blood Flow Velocity	MBFV_HVbl_	average of MBFV values of MCAs for the 10 s preceding HV
Mean Blood Flow Velocity—Minimum Value	MBFV_HVmin_	minimum value of MCA MBFV during HV
Mean Blood Flow Velocity—At the End of 30 sec Hyperventilation	MBFV_HV30_	MCA MBFV value at the end of HV test
Difference in Mean Blood Flow Velocity Values Regarding the HV Test	ΔMBFV_HV_	the difference between MCA MBFV_BL_ and MBFV_HVmin_
Time to the Minimum Value of Mean Blood Flow Velocity During Hyperventilation	time-to-MBFV_HVmin_	is the time passed from the beginning of HV until MCA MBFV_HVmin_
Hyperventilation Index	HVI	HVI = $\frac{\Delta MBFVHV}{MBFVHVbl}\times \frac{1}{30}\;\times 100$
Mean Blood Flow Velocity Values at the Beginning of Breath-Holding	MBFV_BHstart_	MCA MBFV value at the beginning of the BH (same as MBFV_HV30_
Mean Blood Flow Velocity Values at the End of Breath-holding	MBFV_BHend_	MCA MBFV value recorded at the end of the BH
Difference in the Mean Blood Flow Velocity Values Regarding Breath-Holding Test	ΔMBFV_BH_	ΔMBFV_BH_ was calculated by the difference between MCA MBFV_BHend_–MBFV_BHstart_
Time to the Baseline Mean Blood Flow Velocity Values During Breath-Holding	time to MBFV_BHbl_	the time passed from MCA MBFV_BHstart_ until it reaches the baseline MBFV value
Time of Breath-Holding	time of BH	the time passed from MBFV_BHstart_ until MBFV_BHend_
Breath-Holding Index	BHI	BHI = $\frac{\left(MBFVBHend-MBFVBHstart\right)}{MBFVBHstart}\times 100\times \frac{1}{tBH}$ [7]

**Table 2:** Summary of the Cerebral Arterial Resistance values of the Hyperventilation and Breath-Holding test.

Definition of Indicator	Abbreviation	Description
Cerebral Arterial Resistance— Baseline Values	CAR_bl_	average of CAR values for the 10 s preceding HV/BH test
Cerebral Arterial Resistance— Maximal Values	CAR_max_	the maximum CAR value registered during the HV/BH test
Cerebral Arterial Resistance— Minimal Values	CAR_min_	is the minimum CAR value registered during the HV/BH test
Difference in CAR Values Regarding the Maximum and Minimum Phases	ΔCAR	the difference between CAR_max_ and CAR_min_
Time From the Baseline CAR Values Until the Maximal CAR Values	CAR_time-to-max_	the time passed from CAR_bl_ until CAR_maxHV_
Time From the Maximum CAR Value Until the Minimal CAR Value	CAR_time-to-min_	the time passed from CAR_max_ to CAR _min_
Cerebral Arterial Resistance Index During Hyperventilation	CARI_HV_	$CARI-HV=\frac{\left({CAR}_{maxHV}-CARbl\right)}{\left(CARBL\right)}\times$100$\times \frac{1}{30}$
Cerebral Arterial Resistance Index During Breath-Holding	CARI_BH_	$CARI-BH=\frac{\left({CAR}_{min}-{CAR}_{max}\right)}{\left({CAR}_{max}\right)}\times$100$\times \frac{1}{tBH}$

**Table 3:** Summary of the variables defining common carotid artery compression test.

Definition of Indicator	Abbreviation	Description
Return to Baseline	RTB	the time passed from the release of the carotid compression until the MCA velocity values reached its baseline
Transient Hyperemic Response Ratio	THRR	THRR was defined from the MBFV—THRR_MBFV_, and peak systolic velocity (PSV)—THRR_PSV_ values [25]. THRR = $\frac{\left(F3-F1\right)}{F1}\;$, where F1 is the baseline BFV, and F3 means the BFV value just after the release of the compression
Cerebral Arterial Resistance Values—Area Under the Curve During CCC Test	CAR_CCCAUC_:	the cumulative change in CAR until return to baseline CAR was determined by calculating the area under the curve (AUC) using the so-called trapezoidal method mentioned before

**Table 4:** Summary of the variables defining the Valsalva Maneuver.

Definition of Indicator	Abbreviation	Description
Centro-Peripheral Valsalva Ratio	CPRV	reflects the changes regarding the MFBV and MABP between phases 2b and 3 [46]. CPVR = $\frac{MBFV3-MBFV2b}{MABP3-MABP2b}$
Centro-Peripheral Overshoot Index	CPOI	based on the CPVR, we defined the CPOI which refers to the difference between the OS and BL values of the mean BFV, and in the denominator is the difference between the OS and BL values of the MABP CPOI $=\frac{\left(MBFVos-MBFV1\right)}{\left(MABPos-MABP1\right)}$
Cerebrovascular Valsalva Ratio	CVAR	expresses the increase in mean BFV in phase IIb compared to phase III [46,56] CVAR = $\frac{\left(MBFVIII-MBFVIIb\right)}{\left(tIII-tIIb\right)}$
Time to Phase IIb of Valsalva Maneuver	time-to-2b	temporal variables refer to the time elapsed until phase 2b
Time to Phase Overshoot of Valsalva Maneuver	time-to-OS	temporal variables refer to the time elapsed until phase OS
Sympathetic Index	SI	expresses the percentage increase in the MABP measured at the end of phase II (MABP IIend) compared to the initial baseline MABP (MABP BL). Normal value of SI is ≥ 0. Based on the SI, patients with cardiovascular ANS dysfunction were defined with a negative SI value [44,57]. SI = $\frac{\left(MABP\;IIend-MABP\;BL\right)}{MBPBL}$ × 100
Pressure Recovery Time	PRT	is defined as a time interval (in seconds) that starts when the systolic ABP is lowest in phase III (t1) and ends when the systolic ABP reaches the BL value again in phase IV (t2). The normal value of PRT is ˂4 s. Based on the PRT, patients with a PRT longer than four seconds were considered to have cardiovascular ANS dysfunction [17,57]. PRT = t_2_ − t_1_
Valsalva Heart Rate Ratio	VHRR	is defined as the maximum heart rate (HRmax) during the maneuver divided by the lowest heart rate (HRmin) obtained within 30 s after the peak heart rate. The normal value of VHRR over 60 years is >1.35. Patients with a VHRR less than 1.35 were considered having cardiovascular ANS dysfunction [49,57] VHRR = $\frac{HRmax}{HRmin}$

**Table 5:** Summary of differences in the measured parameters and calculated indices of the hyperventilation and breath-holding test of 31 patients.

Variables	ICA_op_ (Mean + SD)	ICA_nonop_ (Mean + SD)	*p* Value
MBFV_HVbl_	52.21 ± 13.86 cm/s	53.54 ± 14.11 cm/s	0.81
MBFV_HVmin_	33.60 ± 9.60 cm/s	33.30 ± 9.80 cm/s	0.69
ΔMBFV_HV_	18.61 ± 7.74 cm/s	20.24 ± 8.19 cm/s	0.6
MBFV_HV30s_	34.72 ± 9.93 cm/s	35.16 ± 9.44 cm/s	0.94
**time to MBFV_HVmin_**	**22.98 ± 5.36 s**	**24.66 ± 5.08 s**	**0.012**
HVI	−1.10 ± 0.32	−1.12 ± 0.34	0.64
MBFV_BHstart_	34.72 ± 9.93 cm/s	35.11 ± 9.48 cm/s	0.97
MBFV_BHend_	60.16 ± 15.99 cm/s	63.75 ± 19.10 cm/s	0.26
ΔMBFV_BH_	25.44 ± 9.77 cm/s	28.65 ± 13.4 cm/s	0.08
time to MBFV_BHbl_	29.42 ± 14.65 s	30.79 ± 14.64 s	0.85
BHI	2.35 ± 1.87	2.63 ± 2.60	0.32
CAR_bl_	2.02 ± 0.59 mmHg×s/cm	1.93 ± 0.54 mmHg×s/cm	0.53
CAR_maxHV_	2.98 ± 1.00 mmHg×s/cm	2.96 ± 1.16 mmHg×s/cm	0.81
CAR_minBH_	1.75 ± 0.51 mmHg×s/cm	1.66 ± 0.50 mmHg×s/cm	0.31
ΔCAR	1.22 ± 0.63 mmHg×s/cm	1.30 ± 0.92 mmHg×s/cm	0.75
CAR_time-to-max_	27.53 ± 11.03 s	27.37 ± 10.19 s	0.43
CAR_time-to-min_	50.05 ± 22.40 s	54.05 ± 22.55 s	0.11
CARI_HV_	1.56 ± 0.93	1.72 ± 1.10	0.29
CARI_BH_	−1.19 ± 0.70	−1.21 ± 0.70	0.43
CAR_HVAUC_	15.75 ± 12.01 mmHg×s/cm	14.45 ± 9.05 mmHg×s/cm	0.8
CAR_BHAUC_	4.05 ± 4.89 mmHg×s/cm	5.00 ± 4.86 mmHg×s/cm	0.36

Values are mean ± SD using Wilcoxon ranked test (*p* < 0.05). Significant differences are indicated in bold letter.

**Table 6:** Summary of differences in the measured parameters and calculated indices in case of 26 patients of the common carotid artery compression test.

Variables	ICA_op_ (Mean + SD)	ICA_nonop_ (Mean + SD)	*p* Value
MBFV_CCCbl_	46.86 ± 16.58 cm/s	52.21 ± 18.19 cm/s	0.16
**PSV_CCCbl_**	**81.55 ± 25.87** cm/s	**94.30 ± 28.11** cm/s	**0.0096**
RTB	19. 35 ± 14.72 s	18.15 ± 12.22 s	0.94
THRR_MBFV_	0.12 ± 0.17	0.20 ± 0.25	0.13
**THRR_PSV_**	**8.80 ± 28.17**	**31.07 ± 29.04**	**0.0046**
CAR_CCCAUC_	**4.46 ± 4.64** mmHg × s/cm	**6.46 ± 5.13** mmHg × s/cm	**0.021**

Values are mean ± SD using Wilcoxon ranked test (*p* < 0.05). Significant differences are indicated in bold letter.

**Table 7:** Summary of differences in the measured parameters and calculated indices of the Valsalva Maneuver (VM) in case of 34 patients.

Variables	ICA_op_ (Mean + SD)	ICA_nonop_ (Mean + SD)	*p* Value
MBFV_VMbl_	52.81 ± 18.21 cm/s	52.14 ± 16.48 cm/s	0.59
CPRV	0.20 ± 7.84	−1.57 ± 5.93	0.5
**CVAR**	**0.91 ± 1.33**	**1.47 ± 1.43**	**0.002**
CPOI	1.57 ± 6.94	1.42 ± 6.87	0.72
time-to-2b	8.18 ± 2.69 s	8.15 ± 2.70 s	0.86
**time-to-OS**	**22.68 ± 3.54 s**	**21.49 ± 2.80 s**	**0.015**

Values are mean ± SD using Wilcoxon ranked test (*p* < 0.05). Significant differences are indicated in bold letters.

## Data Availability

The raw data supporting the conclusions of this article will be made available by the authors on request.

## Abbreviations

ABP	Arterial Blood Pressure
AUC	Area Under the Curve
BFV	Blood Flow Velocity
BH	Breath-Holding Test
BHI	Breath-Holding Index
BL	Baseline
CAR	Cerebral Arterial Resistance
CCA	Common Carotid Artery
CAR-AUC	Cerebral Arterial Resistance—Area Under the Curve
CCC	Common Carotid Artery Compression Test
CEA	Carotid Endarterectomy
CPVR	Centro-Peripheral Valsalva Ratio
CPOI	Centro-Peripheral Overshoot Index
CVAR	Cerebrovascular Valsalva Ratio
CVR	Cerebral Vasoreactivity
ECG	Electrocardiogram
ESVS	European Society for Vascular Surgery
HV	Hyperventilation
HVI	Hyperventilation Index
ICA	Internal Carotid Artery
ICAop	Side to be operated
ICAnonop	Side no to be operated
ICAS	Internal Carotid Artery Stenosis
MABP	Mean Arterial Blood Pressure
MBFV	Mean Blood Flow Velocity
MCA	Middle Cerebral Artery
NASCET	North American Symptomatic Carotid Endarterectomy Trial
PRT	Pressure Recovery Time
PSV	Peak Systolic Velocity
RTB	Return to Baseline
SI	Sympathetic Index
TCD	Transcranial Doppler
THR	Transient Hyperemic Response
THRR	Transient Hyperemic Response Ratio
VM	Valsalva Maneuver
VHRR	Valsalva Heart Rate Ratio
